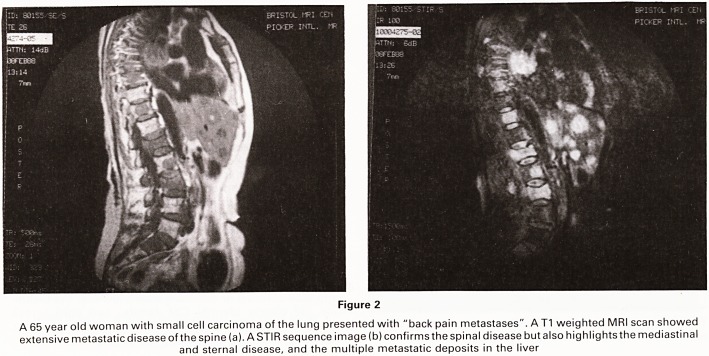# The Detection of Metastatic Disease of the Spine in Oncology Patients Using MRI

**Published:** 1988-05

**Authors:** P. Goddard, I. Watt, E. R. Davies, P. Cook, J. Waring, B. Hale

## Abstract

The value of MRI in the diagnosis and management of patients with primary bone tumours has already been established.

The use of MRI in the detection of metastatic spinal disease is discussed in this paper.


### The Detection of Metastatic Disease of the Spine in Oncology Patients Using MRI

**P. Goddard MD FRCR, I. Watt FRCR, E. R. Davies FRCR, P. Cook MBChB, J. Waring, B. Hale FRCR**

### SUMMARY

The value of MRI in the diagnosis and management of patients with primary bone tumours has already been established.

The use of MRI in the detection of metastatic spinal disease is discussed in this paper.

### INTRODUCTION

Skeletal metastatic disease occurs in 20–35 per cent of patients with a malignancy (1, 2). Lung, breast, genito-urinary and gastro-intestinal tumours account for 80 per cent of primary sites. Breast is the commonest primary in females and prostate the commonest in males. Metastases are usually multiple but solitary deposits occur in approximately 10 per cent. The axial spine is a common site for metastatic disease with up to 52 per cent of metastatic bone disease occurring at this site.

Plain radiographs, radionuclide bone imaging and CT of the spine are all part of the investigation of patients with suspected metastatic disease of the spine. All of these techniques have their limitations however. It has been demonstrated that the loss of 50–75 per cent of cancellous bone is necessary for the detection of autopsy proven metastases on the lateral lumbar spine radiograph. Radionuclide bone imaging is highly sensitive, but not very specific for the detection of metastases and has a high false positive rate, particularly in the isolated lesion. Further, false negatives can occur in some malignancies. Thin section axial CT of the spine can be difficult to interpret and the correct level must be selected as CT of the entire spine is very time consuming. Even then some metastatic disease may be missed.

The limitations of these techniques are compounded in older patients with known malignant disease, where osteopenic fractures (from any of the causes of osteopenia), previous radio-therapy or recurrent malignancy make interpretation even more difficult.

A number of papers have shown MRI to be superior to CT in demonstrating the marrow involvement in primary bone tumours and reticuloses (3, 4, 5). Until recently only scattered cases of metastatic disease of the spine demonstrated by MRI have appeared in the literature. More recently it has been shown that MRI provides excellent anatomical detail in skeletal metastatic disease, enabling accurate biopsy specimens to be obtained (6). Further work has shown that the changes occurring in bone marrow after radiotherapy are characteristic (7). In view of these findings it was felt that MRI would be extremely useful in the differentiation of the various causes of back pain in patients with a known primary tumour, where the plain films had been unhelpful.

### PATIENTS AND METHODS

Thirteen patients presenting with known primary malignant disease, back pain and suspected metastatic disease of the spine were scanned with a Picker VISTA 2055 HP 0.5 Tesla MRI scanner, using body surface coils designed for use in imaging of the spine.

Of the 13 patients 6 were male and 7 female. The age range was from 25 to 86 years with a mean age of 63 years. The primary tumours consisted of breast 5, lymphoma 2, prostate 2, lung 2, teratoma testis 1, antral carcinoma 1.

Plain films were obtained in all cases. CT was obtained in two cases and radionuclide bone scans in a further two cases as part of the 'routine' investigation of these patients.

### Several MRI sequences were used

—T1 weighted sagittal spin echo (TR 500 ms, TE 26 ms)

—STIR short tau inversion recovery (TR 1500 ms, TI 100 ms)

The value for TI for our machine is 100ms to effectively suppress the signal from fat, also increasing tissue contrast due to the additive effects of T1 and T2 components.

—T2 weighted spin echo MAST (TR 2000 ms, TE 100 ms)

—FE Dif (TR 400 ms, Te 20 ms)

FIELD ECHO produces an image enhancing the chemical shift effect of water minus fat (FE DIF).

### Results

The magnetic resonance images showed good anatomical detail in all cases, enabling diagnosis of the cause of the back pain. The images also provided sufficiently accurate anatomical detail to enable palliative radiotherapy to be initially planned in those patients with metastatic disease.

Metastatic or multicentric disease was shown in 12 case (Figure 1). Degenerative disease in addition to metastatic disease was shown in 4 patients and degenerative disease alone was shown in one case. In 3 cases enlarged para-aortic nodes were demonstrated. In one case the left kidney was involved and in a further case the IVC was thrombosed and collateral circulation demonstrated. Figure 2 demonstrates the dramatic contrast between normal soft tissue and malignant disease which can be seen using the STIR sequence, thus providing further information as to the extent of any metastatic disease.

With the sequences used the appearances of metastatic disease of the spine were highly characteristic. On T1 weighted, spin echo images the metastatic disease appeared as dark areas (reduced signal) against a background of white, normal marrow fat. On the STIR sequence, the metastatic deposits appeared as white (high signal intensity) against a background of grey, or black, normal marrow fat. The metastasis-normal tissue contrast was very good and particularly noticeable in the soft tissues where metastatic deposits were easily recognised. On the T2 weighted MAST sequence the metastases and normal marrow were both white or light grey with little contrast or differentiation. However the technique did clearly identify CSF (white) and cord (grey), allowing demonstration of thecal or cord compression, obviating the need for myelography.

**Figure 1**

An 85 year old woman presenting with carcinoma of the breast and an osteopenic lumbar spine (a). The appearances suggested a diagnosis of osteoporosis and associated collapse of L4. A T1 weighted MRI scan showed widespread, well defined lesions throughout the lumbar spine due to metastases (b)

**Figure 2**

A 65 year old woman with small cell carcinoma of the lung presented with "back pain metastases". A T1 weighted MRI scan showed extensive metastatic disease of the spine (a). A STIR sequence image (b) confirms the spinal disease but also highlights the mediastinal and sternal disease, and the multiple metastatic deposits in the liver

**Discussion**

Metastatic deposits were shown as multiple, well-defined focal lesions of reduced signal intensity on the T1 weighted images. These appearances were characteristic and totally different from the appearances due to collapse from osteoporosis or other benign causes. This correlates well with previous reports of metastatic disease of the spine. Both intra- and extra-osseous disease was shown on a single sagittal T1 scan, with a similar signal intensity.

The simplest technique used for showing bony metastatic disease in the spine was the T1 weighted spin echo sequence in the sagittal plane (scan time 9 minutes). When centred on the lumbar spine this method demonstrated the spine from T10 to the coccyx.

For a patient with low back pain this method encompasses most of the region of interest in a single scan.

The Mast T2 sequence (scan time 13 minutes) was also used where cord compression was suspected or equivocal on the T1 weighted images.

The STIR sequence (scan time 13 minutes) provided no further information for the intra-osseous disease but proved extremely useful in the demonstration of extra-osseous disease, where the high tissue contrast enabled the detection of small deposits in lymph nodes, liver and muscle. Anatomical detail was better demonstrated on the T1 scan however.

**Conclusion**

Magnetic resonance is a highly effective and time efficient method of detecting metastatic disease of the spine. The T1 weighted spin echo sequence (TR 500 ms, TE 26 ms) is the method of choice with the STIR and MAST T2 sequences used as adjuncts.

**ACKNOWLEDGEMENTS**

The authors wish to thank Miss Ann Case, Dr B. Penry, Dr C. Johnson, Dr G. Thompson, Mrs Gloria Hatton and Mrs Rona Wright for their assistance in the Bristol MRI Centre and Dr J. Bullimore, Dr E. Whipp, Dr V. Barley and Dr G. Rees for referring patients.

**REFERENCES**

1. ABRAMS, H. L. SPRIO, R. GOLDSTEIN N (1950) Metastases in carcinoma: an analysis of 1000 autopsied cases. *Cancer* **3**, 74–85.
2. TURNER, J. W. JAFFE, H. L. (1940) Metastatic neoplasms: a clinical and roentgenological study of involvement of skeleton and lungs. *AJR* **43**, 479–492.
3. RICHARDSON, M. L. KILCOYNE, R. F. GILLESPY, T. HELMS, C. A. GENANT H. K. (1986) Magnetic resonance imaging of musculoskeletal neoplasms. *Radiol Clin North Am* **24**, 259–267.
4. BOHNDORF, K. REISER, M. LOCHNER, B. DE LACROIX, W. F. STEINBRICH, W. (1986) Magnetic resonance imaging of primary tumours and tumour-like lesions of bone. *Skeletal Radiol* **15**, 511–517.
5. AISEN, A. M. MARTEL, W. BRAUNSTEIN, E. M. MCMILLAN, K. I. PHILLIPS, W. A. KLING, T. F. (1986) MRI and CT evaluation of primary bone and soft tissue tumours. *AJR* **146**, 740–756.
6. DAFFNER, R. H. LUPETIN, A. R. DASH, N. DEEB, L. SEFCZEK, R. J. SCHAPIRO, R. L. (1986) MRI in the detection of malignant infiltration of bone marrow. *AJR* **146**, 353–358.
7. RAMSEY, R. G. ZACHARIAS, C. E. (1985) MR imaging of the spine after radiation therapy: easily recognisable effects. AJR 1985; 144: 1131–1135. *AJNR* **6**, 247–251.

## Figures and Tables

**Figure 1 f1:**
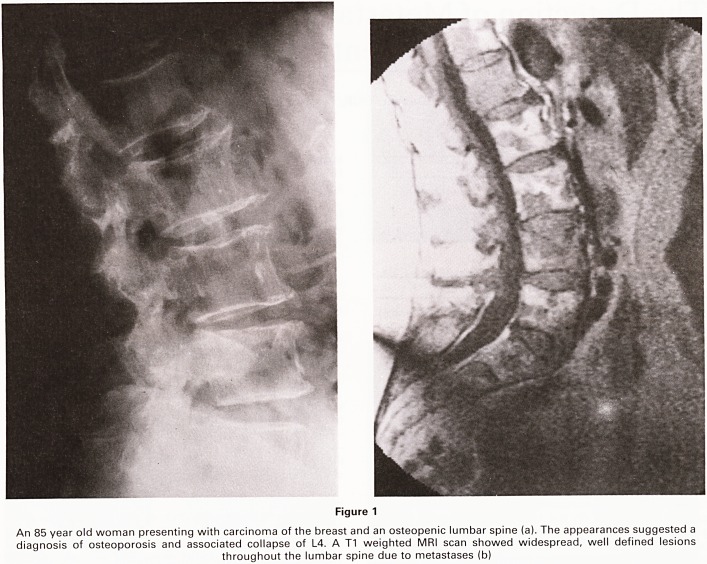


**Figure 2 f2:**